# The Influence of Urbanization Modes on the Spatial Circulation of Flaviviruses within Ouagadougou (Burkina Faso)

**DOI:** 10.3390/ijerph13121226

**Published:** 2016-12-10

**Authors:** Florence Fournet, Stéphane Rican, Zoé Vaillant, Anna Roudot, Aude Meunier-Nikiema, Daouda Kassié, Roch K. Dabiré, Gérard Salem

**Affiliations:** 1 Unité Mixte de Recherche Maladies Infectieuses et Vecteurs: Ecologie, Génétique, Evolution et Contrôle (MIVEGEC), Institut de Recherche pour le Développement, Montpellier 34394, France; 2 Institut de Recherche en Sciences de la Santé/Centre Muraz, Bobo-Dioulasso BP 545, Burkina Faso; dabire_roch@hotmail.com; 3 Laboratoire Dynamiques Sociales et Recomposition des Espaces (LADYSS), Université Paris Ouest Nanterre La Défense, Nanterre 92000, France; srican@u-paris10.fr (S.R.); zoe.vaillant@u-paris10.fr (Z.V.); annaroudot@gmail.com (A.R.); daouda.kassie@cirad.fr (D.K.); salem.gerard@gmail.com (G.S.); 4 Institut des Sciences des Sociétés, Ouagadougou BP 7047, Burkina Faso; nikiaude@yahoo.fr; 5 Unité Recherche Animal et Gestion Intégrée des Risques (AGIRS), Centre de Coopération Internationale en Recherche Agronomique pour le Développement, Montpellier 34398, France; 6 Unité Mixte de Recherche Centre Population et Développement (CEPED), Institut de Recherche pour le Développement, Paris 75006, France

**Keywords:** West Africa, Burkina Faso, urban health, health inequalities, infectious diseases, arboviruses

## Abstract

Dengue is an emerging infectious disease of global significance. Although this virus has been reported for a long time, its significance within the burden of diseases in West Africa is not obvious, especially in Burkina Faso. Our objective was to evaluate flavivirus presence in Ouagadougou (Burkina Faso) and the link between anti-flavivirus antibody seroprevalence and urbanization modes. A population-based cross-sectional survey was conducted and 3015 children were enrolled from Ouagadougou districts with different types and degrees of urbanization (with/without equipment and high/low building density). Flavivirus (FLAV) IgM MAC-ELISA and FLAV indirect IgG ELISA were performed. Associations between FLAV IgG presence (sign of past infection) and various independent variables were assessed using the chi-square test and a multivariate logistic regression analysis. The apparent prevalence of past flavivirus infections among the enrolled children was 22.7% (95% CI: 22.4–26.7) (*n* = 685). Eleven children (0.4%; 95% CI: 0.61–2.14) were positive for FLAV IgM, indicating active transmission. Factors associated with flavivirus infection were identified among the enrolled children (age, sex), householders (educational level, asset index) and in the environment (building density, water access, waste management and house appearance); however, they showed great variability according to the city districts. The water access modality did not significantly influence FLAV IgG positivity. Conversely, apparently good practices of waste management had unexpected consequences (increased risk related to municipal dumpsters). Given the scale of ongoing urbanization and the spread of arboviral diseases, close collaboration between health and city stakeholders is needed.

## 1. Introduction

Malaria has captured the attention of the research and medical communities since its description as the first proof of a vector-borne disease before plague, dengue or yellow fever. However, the development of arbovirology research [1] and some recent studies suggest that malaria clinical presumption may be overestimated and that a significant proportion of febrile diseases could be caused by arboviruses, such as dengue, chikungunya, or yellow fever virus [2,3,4]. Despite yellow fever mass vaccination campaigns to prevent and control outbreaks, the risk of epidemics has greatly increased, especially in poor and densely populated urban settings, both in Africa and South America [5,6].

In 2008, 16 yellow fever outbreaks were reported from 13 African countries, reflecting an intense virus circulation across the western and central parts of Africa [6]. Consequently, yellow fever is considered as an emerging or reemerging disease of considerable importance. Furthermore, since 2000, dengue cases have been described in several West and Central Africa countries: Burkina Faso (2007, 2008, 2013–2015), Cameroon (2006), Ivory Coast (2007, 2008, 2010), Gabon (2007, 2008, 2010), Mali (2008) and Senegal (2000, 2007–2009) [3,7,8,9]. In 2009, a large outbreak of dengue fever (more than 6000 cases) occurred in the Cape Verde Islands, where dengue virus was never isolated before, leading to considering the phenomenon as a true emergence of dengue fever in Africa [10].

Such a dramatic increase in arboviral diseases could be linked to different events, among which unplanned urbanization plays a crucial role. For instance, larvae of the dengue vector *Aedes aegypti* breed in a variety of human-generated containers, such as jars, discarded cans, flower vases, cement tanks, ant traps, used tires and plastic buckets around human dwellings. Furthermore, *Ae. aegypti* diurnal and strongly anthropophilic behavior might promote pathogen transmission [11].

In Burkina Faso, the epidemiological situation of arboviruses is currently poorly documented. The first dengue fever outbreak was described in the 1980s [12] and in the following years several limited outbreaks occurred in Ouagadougou and Bobo-Dioulasso, the country’s main towns. In 2013, dengue was reported in healthcare centers of Ouagadougou [9]. For yellow fever, the survey carried out by Yaro et al. [13] in the Southwestern part of Burkina Faso showed an increase of confirmed cases between 2003 and 2005, possibly associated with the intensification of migration between Burkina Faso and Côte d’Ivoire.

Therefore, with the research project “Urban environment and health transition in West Africa: the example of Ouagadougou (Burkina Faso)”, we wanted to analyze the spatial distribution of communicable and non-communicable diseases, by taking into account Ouagadougou urbanization pattern [14,15,16]. The objectives of the flavivirus study were to evaluate their presence in Ouagadougou and to explore the link between flaviviruses and urbanization. Our hypothesis was that flavivirus circulation is heterogeneous due to the differentiated urbanization process, leading to the diversification of life environments, water storage and waste management practices among Ouagadougou inhabitants.

## 2. Methods

### 2.1. Study Area

Ouagadougou (12°21′14″ N, 1°30′41″ W) is the capital city of Burkina Faso, one of the poorest nations in the world and one of the less urbanized countries of the West African sub-region [17]. Ouagadougou has a savannah climate with a mean annual rainfall of 935 mm and a mean temperature of 28 °C. The dry season extends from November to April. In 1996, city-dwellers hardly represented 20% of the total population of Burkina Faso [18] compared, for example, with 50% in Côte d’Ivoire [17]. In 2004, year of the present serological survey, almost half of Burkina Faso’s urban population lived in Ouagadougou. The capital city population increased from 282,000 inhabitants in 1985, to 709,000 inhabitants in 1996 and to 1,200,000 inhabitants in 2004, concomitantly with spectacular spatial growth. In 2003, Ouagadougou extended over more than 200 km^2^, compared with an estimated 33 km^2^ after the independence in 1960 [19]. Since the revolution of 1983, significant efforts have been made by the municipality to develop public services (water, electricity, road network, health services, schools, etc.). However, unplanned urbanization has continued and, in 2004, 44% of Ouagadougou remained unplanned and consequently without urban facilities. Moreover, because of the specific water policy imposed by Sankara during the revolution in the 1980s, 55.3% of households use public fountains for water supply and, as a consequence, most of them store water within their house [19,20].

### 2.2. Sampling Strategy

**Figure 1:**
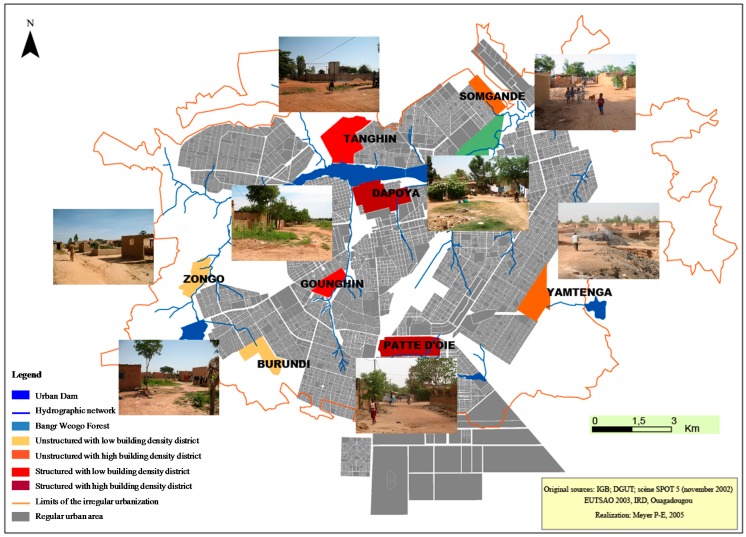
Study area showing the different districts within Ouagadougou city.

Ouagadougou city was stratified by identifying and selecting ecological and environmental situations that were representative of the urbanization process. The first stratification criterion was the allotment status: structured areas compared with unstructured areas, as a representation of the vulnerability of the population living at the urbanization front. Structured areas were areas allotted by the township authority (cadastral services) with public services, such as tap water and sanitation, electricity and telephone, and where parcels of land were allocated to specific inhabitants. Unstructured areas referred to areas that developed without cadastral organization and without public services. The second stratification criterion was the building density that represents a risk of infectious disease transmission because of the high promiscuity in areas with high density housing. Stratification was carried out using a SPOT^®^5 remote sensing scene obtained in November 2002 with a resolution of 2.5 m as well as aerial photographs of Ouagadougou and cadastral data from the Ministry of Urbanization of Burkina Faso [21]. These spatial data were confirmed by field observations. Altogether, four strata were determined: unstructured with low building density (ULBD), unstructured with high building density (UHBD), structured with low building density (SLBD), and structured with high building density (SHBD). In each stratum, two districts were selected for the study; thus, a total of eight districts were included: Zongo and Burundi in the ULBD stratum, Somgande and Yamtenga in UHBD stratum, Tanghin and Gounghin in SLBD stratum, Dapoya and Patte d’Oie in SHBD stratum (Figure 1).

### 2.3. Sample Design and Studied Population

In structured districts, 250 parcels were randomly selected using cadastral data and a generated set of random numbers. In unstructured districts, 250 starting points were selected using aerial photographs of Ouagadougou and pairs of generated random numbers that corresponded to general positioning system (GPS) data points. Each pair of data points was identified on the photographs. If they corresponded to a household, this household served as starting household and the next three eligible households were included in the study. Households were considered eligible if the householder had resided in Ouagadougou for at least five years, a period of time that ensured a sufficient exposure to the urban lifestyle [22,23]. We assumed that all the people living with him/her had the same exposure time to the urban environment.

In each eligible household, all children from six months to twelve years of age were invited to a mobile health post with an accompanying family adult for data collection. In low-income countries, children under the age of five are particularly vulnerable to infectious diseases, such as respiratory infections or malaria. On the other hand, dengue is more likely to occur in children under 15 years of age [24]. We focused on children younger than 12 years because we considered that up to this age they are not very mobile outside their neighborhood, and therefore, that they could be considered as good indicators of flavivirus circulation within each district.

### 2.4. Data Collection

Sample collection and interviews were carried out between May and September 2004. Standardized questionnaires were administered to collect information on the householder (educational level, residence duration in Ouagadougou) and his/her children (age, sex, immunization against yellow fever according to the health record). Environmental data (compound geographic coordinates; urban variables, such as land tenure and building density of the district, as well as water access, waste management and house appearance) were also recorded when visiting the household.

Children were divided into three age groups: 0–4 years, 5–10 years and 11–12 years. An asset index and an education level index were calculated for each family. The asset index (high, medium and low) was determined on the basis of the possession of assets and is a proxy of the socio-economic status. Telephone (mobile or not), television, refrigerator, ventilator, bed, living room and motorcycle were considered as assets. The education level was estimated by asking whether the householder attended school, whatever the duration. The householder was also interviewed about water access according to three modalities (tap water, pump or water well) and about waste management (considered as “improper”, if burned in the environment or if collected in municipal dumpsters that were not regularly emptied or “adequate”, if collected by a private service). The house appearance was assessed and ranked as “good” or “not good”, according to the care given by the household to the house environment. 

At the mobile health post, blood samples of 25 µL were collected by fingerpick from the children and absorbed on filter papers (Whatman^®^, Proteinsaver^TM^) for subsequent analysis, as previously described [25]. Filter papers were stored at room temperature for one month until they were sent to the laboratory (Center for Vectors and Vector Diseases, Mahidol University, Bangkok, Thailand), where they were stored at −80 °C until analysis.

### 2.5. Laboratory Analysis

Blood samples blotted on filter paper were cut out using a 0.5 cm diameter paper punch. For each sample, two discs were eluted in 800 µL of phosphate-buffered saline (PBS) buffer 5% non-fat dried milk. After vortexing for 60 s, samples were incubated at room temperature for one hour, then vortexed again and centrifuged for 10 min (10,000 rpm, 4 °C). The recovered supernatants corresponded to a 1:100 dilution of the original blood samples. Flavivirus IgM MAC-ELISA and indirect IgG ELISA assays were performed as previously described [26], using sucrose-acetone extracted viral antigens, as developed by the Center for Vaccine Development (Mahidol University, Bangkok, Thailand), and commercially available mouse anti-virus monoclonal antibodies [27]. For IgM detection, samples were considered positive when the Differential Optical Density (DOD) value was >0.50. For IgG, samples with a DOD value >2.00 were considered positive. For quality control, a positive control and three negative controls were always included in each plate, and specimens collected at different times were simultaneously tested. The cut-off OD value for positive samples was equal to the mean OD value of three negative controls plus three standard deviations. To be considered positive, the sample OD needed to be higher than this cut-off value. Samples positive for anti-flavivirus IgM (thereafter, “FLAV IgM”) were scored as “recent flavivirus infections”. Serum samples negative for FLAV IgM and positive for anti-flavivirus IgG (thereafter, “FLAV IgG”) were considered as “past flavivirus infections”. 

### 2.6. Statistical Analysis

Data were entered in a Microsoft Access 2000 database (Palisade Corp., Newfield, NY, USA), and analyzed using SPSS 12.0 for Windows (SPSS Inc., Chicago, IL, USA).

The dependent variable was FLAV IgG apparent prevalence that represents past exposure to flaviviruses. FLAV IgG apparent prevalence was analyzed relative to the socioeconomic, demographic, health and environmental data concerning the children, their family/household or the district. 

First, the characteristics of the study sample were described and then frequency distributions were used to highlight the status of children positive or not for FLAV IgG. Then, chi-square tests were used to detect statistically significant associations between FLAV IgG presence and various independent variables. Variables associated with FLAV IgG with a *p*-value < 0.20 in bivariate analyses were then considered for the logistic regression analysis [28]. Finally, a multivariate logistic regression analysis was performed in each stratum to estimate the Odds Ratios of the independent variables that explain FLAV IgG presence. Estimates are presented with 95% confidence intervals.

The apparent prevalence of the householder’s age, sex and educational status was calculated at the level of the district to better analyze differences within districts.

FLAV IgG and IgM data in structured districts were also analyzed according to the position of municipal fixed dumpsters that have been set up in the town center since 2000 [29].

### 2.7. Ethics

The research protocol was validated by the Ethics Committee for Research in Health of Burkina Faso (Registration Number No. 2004/002). The mother, father or legal guardian of each child formally agreed to his/her participation in this study after being adeqautely informed of the study and before inclusion.

## 3. Results

A total of 3015 children were included in the survey. Table 1 shows the frequencies of the variables previously reported as potential factors associated with flavivirus transmission and clearly emphasizes the important intra-urban heterogeneity of our sample.

### 3.1. Anti-Flavivirus Antibody Seroprevalence

The apparent prevalence of past flavivirus infections (i.e., percentage of children positive for FLAV IgG, but negative for FLAV IgM) among the survey participants was 22.7% (95% CI: 22.4–26.7) (*n* = 685) (Table 2).

Eleven children (0.4%; 95% CI: 0.61–2.14) were positive for FLAV IgM, indicating active transmission.

FLAV IgG-positive samples were found among children as young as six months and the seroprevalence increased with age, from 11.8% in the 0–4-year-old group to 36.4% in the 11–12-year-old group (*p* < 0.001). Seroprevalence was also significantly higher among girls than boys (*p* < 0.015).

FLAV IgG seroprevalence was not influenced by the duration of the householder’s installation in town, his/her educational level or the house appearance. Conversely, it was significantly higher in medium socioeconomic level households (*p* < 0.003). Improper waste management, water supply from a water well and water storage significantly increased FLAV IgG seroprevalence. Similarly, FLAV IgG was more frequent in the ULBD and SLBD strata than in the other urban strata. However, except for the SHBD stratum, disparities were observed within the different strata. Indeed, FLAV IgG seroprevalence greatly varied within districts of the same stratum. For instance, it doubled between Somgande and Yamtenga (UHBD), between Zongo and Pissy (ULBD) and between Gounghin and Tanghin (SLBD).

### 3.2. Individual and Environmental Factors Associated with Flavivirus Seroprevalence

The multivariate analyses conducted in each stratum highlighted a significant (*p <* 0.001) and positive association of FLAV IgG positivity with age in all strata (Table 3). A positive association for girls was found only in the SHBD stratum, but the tendency was the same in the other strata with the exception of UHBD. The lack of householder’s education tended to increase the chance of FLAV IgG positivity, but the association was significant (*p <* 0.01) only in the ULBD stratum. A medium socioeconomic level seemed to be associated with the presence of FLAV IgG, but this association was significant (*p <* 0.01) only in structured districts (SHBD and SLBD strata).

The association of type of water access with FLAV IgG prevalence was confusing because of the high variations among and within strata. Improper waste management practices were significantly associated with FLAV IgG presence in the UHBD and SHBD strata (respectively *p <* 0.05 and *p <* 0.01). A good house appearance was associated with FLAV IgG positivity in the ULBD stratum (*p <* 0.001).

In the Gounghin district, where FLAV IgG seroprevalence was the highest, FLAV IgG positivity was significantly higher (*p <* 0.05) in households located within 200 m from a dumpster (43%) than far away (31.5%).

## 4. Discussion

In Ouagadougou, 22.7% of children from six months to 12 years of age showed a past flavivirus infection. In Garoua, a town in Northern Cameroon that is also characterized by a savannah climate and where inhabitants traditionally store water inside and around their dwellings in terracotta jars, Demanou et al. [30] reported comparable values (23%) for past dengue infection prevalence. Together, these results are in favor of an active and heterogeneous flavivirus circulation in Ouagadougou as we hypothesized, and suggest that the differential diagnosis of malaria should be improved.

Our study shows a positive association between age and increasing FLAV IgG seroprevalence, as expected for flavivirus transmission in endemic areas [31]. Girls were more affected than boys, as previously reported [32]. This suggests that human-vector contacts might preferentially occur in peri-domestic environments. Indeed, in Burkina Faso, schooling is less widespread among girls than boys and girls tend to stay at home [33]. Moreover, lack of householder’s education was associated with past flavivirus infection, as often observed in the health field [34]. Similarly, the absence of links between flavivirus seroprevalence and socio-economic level confirmed previous results [35]. 

**Figure 2:**
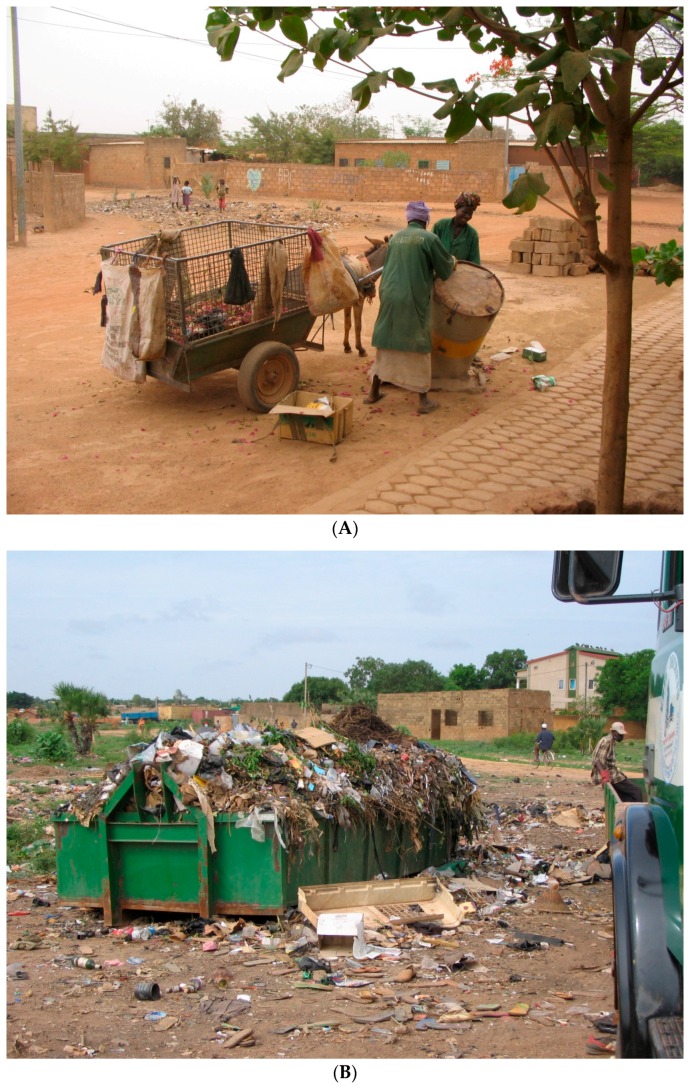
Photographs showing different waste collection modalities. (**A**) Households can start a monthly subscription with an association; here, two women are collecting waste in Gounghin with a donkey car; (**B**) Dumpster full of waste in a street of Gounghin, allowing *Aedes* mosquitoes to develop during the first rainfall.

The association between waste management practices and past flavivirus infection in the different districts of Ouagadougou was consistent with other studies [36,37]. The presence of waste in the environment is frequently considered to be a significant risk factor because empty plastic containers are suitable peri-domestic breeding sites for *Ae. aegypti* during the rainy season. In Ouagadougou, environmental concerns were not shared by the entire population because of the traditional perception of garbage as a gift [38] and also because of the limited concern for public spaces. Although the municipality is committed to developing procedures for waste handling and disposal, the city hall technical services find it difficult to comply with these new procedures. Dumpsters are not regularly emptied, thus providing breeding sites for flavivirus vectors (see photographs in Figure 2).

On the other hand, the finding that FLAV IgG seroprevalence was not influenced by the type of water supply was unexpected due to the importance of water sources for vector breeding [39]. Two hypotheses might explain this. Water is rare in Ouagadougou and, consequently, it is not stored for a long time and containers are regularly emptied. Alternatively, containers could be well covered, thus limiting the possibility of colonization by mosquitoes. Both behaviors have been already observed in North Cameroon [40].

**Figure 3:**
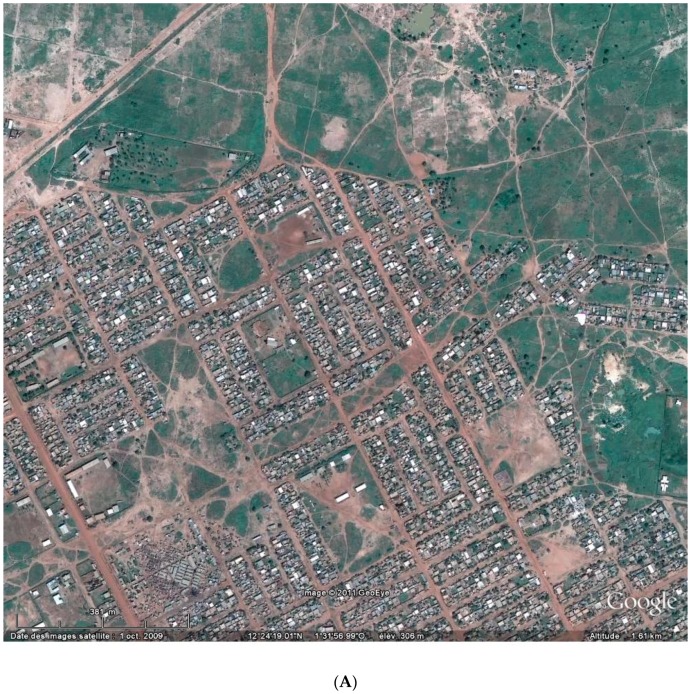

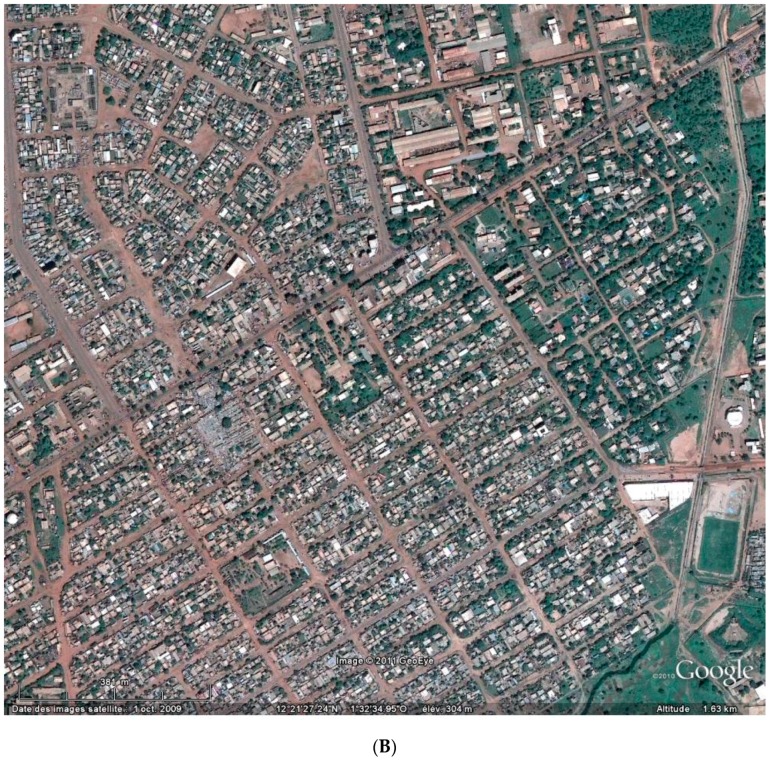
Aerial view of two districts of the study area in 2010 (Google Earth^®^). (**A**) Tanghin with a lot of land reserves; and (**B**) Gounghin with a high building density and no land reserve.

Neglected house appearance, which suggests a lack of consideration for the peri-domestic environment, was associated with a higher chance of infection in low, but not in high building density districts, although several studies reported that high building density can support the presence of *Ae. aegypti* [41,42]. High-rise buildings do not induce the same circulation than low-rise ones. Moreover, the presence of vegetation may also play a role in the vector spatial distribution pattern and consequently in the transmission of the associated flavivirus [43]. This is clearly confirmed by the FLAV IgG variability within the same stratum: high FLAV IgG prevalence was observed in Gounghin (37.3%), but not in Tanghin (19.9%), although both districts belong to the same SLBD stratum. Such differences may be explained by their spatial urbanization structure: house clusters in Gounghin led to higher local building density than in Tanghin where fallow areas are more regularly dispersed (Figure 3). Moreover, municipal garbage dumpsters were installed in Gounghin, but not in Tanghin.

## 5. Limitations of the Study

Due to the retrospective nature of this survey, some of the identified associations could be difficult to interpret. The multivariate analyses did not fully explain the seroprevalence of past flavivirus infections observed in the various districts of Ouagadougou included in this study. The analyses did not identify the variables with direct and indirect influences on the outcome and the variables that can act as confounding factors. This underlines the complexity of this pathogenic system and the multifactorial influences (environmental and also anthropogenic) involved.

Dengue virus and yellow fever virus may be responsible for the FLAV IgG seroprevalence observed in Ouagadougou; however, other flaviviruses are present in this sub-region, for instance Zika virus [44], although they have not yet been reported in Burkina Faso. The yellow fever vaccine is affordable and cost-effective; however, low immunization coverage has been reported in endemic areas, including several countries of South America and Africa. Our study shows that 356 six-month- to six-year-old children were vaccinated (21.3%) among the 1673 children with a health record, indicating a limited coverage. Indeed, between 2000 and 2008, yellow fever cases have been identified in the southeast of Burkina Faso, but not in or around Ouagadougou [13]. Dengue fever cases have been reported in Ouagadougou since 2008 [8,45]. Additional analyses are needed to differentiate past dengue virus infections from past yellow fever virus infections, particularly the plaque reduction neutralization test that is currently considered to be the “gold standard” [46]. This kind of analysis was not within the scope of this study. 

## 6. Conclusions

The uneven distribution of potential risk factors (such as age, sex, household equipment and waste management practices) led to major intra-urban differences in flavivirus circulation in Ouagadougou. Apparently good practices of waste management might have unexpected consequences (increased chance of infection related to municipal dumpsters), and environmental education must become an important public health goal. Due to the intensity of worldwide trade, the growing urbanization in Africa, especially in Burkina Faso, and the difficulty for the local healthcare system to deliver a precise diagnosis based on laboratory test results, it is crucial to improve our knowledge about flavivirus diseases and their clinical presentation. There is also a strong need for better cooperation between urban and health policy makers to improve the health status and well-being of the populations.

## Figures and Tables

**Table 1:** Characteristics of the study population in the four strata.

Category	Variable		UHBD	ULBD	SHBD	SLBD	Total
Child	Sex	Male	448 (51.2) *	422 (51.3)	286 (49.1)	351 (47.8)	1485 (49.3)
		Female	427 (48.8)	400 (48.7)	297 (50.9)	384 (52.2)	1530 (50.7)
	Age	0–4 years	328 (37.5)	296 (36.0)	207 (35.5)	230 (31.3)	1061 (35.2)
		4–10 years	434 (49.6)	406 (49.4)	266 (45.6)	367 (49.9)	1473 (48.9)
		10–12 years	113 (12.9)	120 (14.6)	110 (18.9)	138 (18.8)	481 (16.0)
Householder	Householder Education	Yes	359 (41.0)	427 (51.9)	363 (62.3)	343 (46.7)	1492 (49.5)
		No	516 (59.0)	395 (48.1)	220 (37.7)	392 (53.3)	1523 (50.5)
	Socioeconomic level	Low	656 (75.0)	603 (73.4)	190 (32.6)	373 (50.7)	1822 (60.0)
		Medium	187 (21.4)	197 (24.0)	103 (17.7)	200 (27.2)	687 (23.0)
		High	32 (3.6)	22 (2.6)	290 (49.7)	162 (22.1)	506 (17.0)
Environment	Waste management	Improper	281 (32.1)	304 (37.0)	103 (17.7)	302 (41.1)	1517 (50.3)
		Adequate	594 (67.9)	518 (63.0)	480 (82.3)	433 (58.9)	1498 (49.7)
	Water supply	Tap	37 (4.2)	17 (2.1)	331 (56.8)	240 (32.6)	625 (20.7)
		Pump	766 (87.5)	639 (77.7)	240 (41.2)	465 (63.3)	2110 (70.0)
		Well	72 (8.3)	166 (20.2)	12 (2.0)	30 (4.1)	280 (9.3)
	House appearance	Good	162 (18.5)	347 (42.2)	229 (39.3)	170 (23.1)	908 (30.1)
		Not good	713 (81.5)	475 (57.8)	354 (60.7)	565 (76.9)	2107 (69.9)
	Total		875	822	583	735	3015

UHBD = Unstructured High Building Density; ULBD = Unstructured Low Building Density; SHBD = Structured High Building Density; SLBD = Structured Low Building Density; * *n* (percentage).

**Table 2:** Apparent prevalence of previous flavivirus infections according to individual, household and environmental features.

Independent Variables		%FLAV IgG + (95% CI)	*p*
Sex *	Male	20.7 (22.4–26.7)	**0.015**
	Female	24.4 (18.7–22.8)	
Age *	0–4 years	11.8 (9.8–13.7)	**<0.001**
	4–10 years	25.9 (23.7–28.2)	
	10–12 years	36.4 (32.3–40.9)	
Education of the householder *	Yes	21.6 (19.5–23.7)	**0.15**
	No	24.3 (22.0–26.6)	
Duration of residence of the householder	<10 years	27.4 (21.6–33.3)	0.41
	10 to 20 years	22.6 (19.4–25.7)	
	≥20 years	22.2 (20.4–23.9)	
Socioeconomic level *	Low	22.4 (20.5–24.4)	**0.003**
	Medium	26.5 (23.2–29.8)	
	High	18.2 (14.8–21.5)	
Water supply *	Tap	22.2 (19.0–25.5)	**0.03**
	Pump	21.9 (20.2–23.7)	
	Well	28.9 (23.6–34.2)	
Waste management *	Improper	24.1 (22.0–26.3)	**0.05**
	Adequate	21.2 (19.1–23.2)	
House appearance *	Good	24.4 (20.1–23.6)	**0.12**
	Not good	21.9 (21.7–27.2)	
Strata	UHBD	20.2 (17.6–22.9)	**<0.001**
	ULBD	25.0 (22.1–28.0)	
	SHBD	16.8 (13.6–19.7)	
	SLBD	27.8 (24.4–30.9)	
Districts	Somgandé	29.7 (25.2–34.3)	**<0.001**
	Yamtenga	12.7 (9.6–15.5)	
	Zongo	31.2 (26.6–35.5)	
	Pissy	18.8 (15.2–22.8)	
	Dapoya	15.5 (11.7–19.5)	
	Patte d’Oie	18.4 (13.3–22.7)	
	Gounghin	37.3 (31.7–42.1)	
	Tanghin	19.9 (15.9–23.7)	
Building density	High	18.9 (16.8–20.8)	**<0.001**
	Low	26.3 (24.1–28.5)	
Area structuration	Yes	23.1 (20.5–25.0)	0.97
	No	22.5 (20.6–24.6)	
Total		22.7 (21.2–24.1)	

UHBD = Unstructured High Building Density; ULBD = Unstructured Low Building Density; SHBD = Structured High Building Density; SLBD=Structured Low Building Density. * Variables introduced in the logistic regression analysis.

**Table 3:** Factors associated with flavivirus seroprevalence.

Variables		UHBD	ULBD	SHBD	SLBD
Age	0–4 years	1	1	1	1
	4–10 years	6.52 (3.85–11.01) ***	2.64 (1.76–3.96) ***	1.91 (1.10–3.31) **	1.64 (1.10–2.45) ***
	10–12 years	11.09 (5.97–20.58) ***	5.52 (3.32–9.18) ***	2.72 (1.45–5.11) ***	2.71 (1.69–4.38) ***
Sex	Male	1	1	1	1
	Female	0.92 (0.64–1.30)	1.19 (0.85–1.67)	1.83 (1.15–2.90) *	1.25 (0.90–1.74)
Education of the householder	Yes	1	1	1	1
	No	1.39 (0.95–2.01)	1.44 (1.01–2.05) **	1.17 (0.71–1.92)	1.23 (0.88–1.71)
Socioeconomic level	High	1	1	1	1
	Medium	0.64 (0.24–1.66)	1.65 (0.49–5.53)	1.83 (0.99–3.39) *	2.01 (1.21–3.33) **
	Low	0.47 (0.19–1.18)	1.03 (0.39–4.31)	1.20 (0.65–2.21)	1.50 (0.91–2.47)
Water supply	Tap	1	1	1	1
	Pump	0.55 (0.25–1.22)	7.39 (0.93–58.07) *	0.76 (0.45–1.32)	0.84 (0.57–1.24)
	Well	0.84 (0.32–2.23)	10.27 (1.28–82.41) **	1.34 (0.31–5.75)	1.22 (0.52–2.83)
Waste management	Adequate	1	1	1	1
	Improper	1.56 (1.05–2.34) *	1.17 (0.82–1.66)	1.92 (1.11–3.34) **	0.90 (0.62–1.30)
House appearance	Good	1	1	1	1
	Not good	0.79 (0.49–1.29)	2.16 (1.53–3.04) ***	0.56 (0.45–1.45)	1.02 (0.68–1.53)

UHBD = Unstructured High Building Density; ULBD = Unstructured Low Building Density; SHBD = Structured High Building Density; SLBD = Structured Low Building Density. * *p <* 0.05; ** *p <* 0.01; *** *p <* 0.001.

## References

[1] Downs W.G. (1975). Malaria: The Great Umbrella. Bull. N. Y. Acad. Med..

[2] Franco L., Di Caro A., Carletti F., Vapalahti O., Renaudat C., Zeller H., Tenorio A. (2010). Recent expansion of dengue virus serotype 3 in West Africa. Eurosurveillance.

[3] Jentes E.S., Robinson J., Johnson B.W., Conde I., Sakouvougui Y., Iverson J., Beecher S., Bah M.A., Diakite F., Coulibaly M. (2010). Acute Arboviral Infections in Guinea, West Africa, 2006. Am. J. Trop. Med. Hyg..

[4] Stoler J., al Dashti R., Anto F., Fobil J.N., Gordon A., Awandare G.A. (2014). Deconstructing “malaria”: West Africa as the next front for dengue fever surveillance and control. Acta Trop..

[5] World Health Organization (2010). Yellow fever surveillance and outbreak response: Revision of case definitions, October 2010. Wkly. Epidemiol. Rec..

[6] World Health Organization (2010). Yellow fever in the WHO African and American Regions. Wkly. Epidemiol. Rec..

[7] World Health Organization (2008). DengueNet: Global Surveillance of Dengue and Dengue Haemorrhagic Fever.

[8] Département International, Institut de Veille Sanitaire (InVS) (2011). Bilan Epidémiologique et Grande Tendance, Dengue.

[9] Ridde V., Agier I., Bonnet E., Carabali M., Dabire K.R., Fournet F., Ly A., Meda I.B., Parra B. (2016). Presence of three dengue serotypes in Ouagadougou (Burkina Faso): Research and public health implications. Infect. Dis. Poverty.

[10] World Health Organization (2009). Dengue in Africa: Emergence of DENV-3, Côte d’Ivoire, 2008. Wkly. Epidemiol. Rec..

[11] Higa Y. (2011). Dengue Vectors and their Spatial Distribution. Trop. Med. Health.

[12] Gonzalez J.P., Du Saussay C., Gautun J.C., McCormick J.B., Mouchet J. (1985). La dengue au Burkina Faso (ex Haute-Volta): Epidémies saisonnières en milieu urbain à Ouagadougou. Bull. Soc. Pathol. Exot..

[13] Yaro S., Zango A., Rouamba J., Diabaté A., Dabiré R., Kambiré C., Tiendrebeogo S.M.R., Yonli T., Ouango J.G., Diagbouga S.P. (2010). Situation épidémiologique de la fièvre jaune au Burkina Faso de 2003 à 2008. Bull. Soc. Pathol. Exot..

[14] Niakara A., Fournet F., Gary J., Harang M., Nebie L.V.A., Salem G. (2007). Hypertension, urbanization, social and spatial disparities: A cross-sectional population-based survey in a West African urban environment (Ouagadougou, Burkina Faso). Trans. R. Soc. Trop. Med. Hyg..

[15] Ouedraogo H.Z., Fournet F., Martin-Prével Y., Gary J., Henry M.C., Salem G. (2008). Socio-spatial disparities of obesity among adults in the urban setting of Ouagadougou, Burkina Faso. Public Health Nutr..

[16] Baragatti M., Fournet F., Henry M.C., Assi S., Ouedraogo H., Rogier C., Salem G. (2009). Social and environmental malaria risk factors in urban areas of Ouagadougou, Burkina Faso. Malar. J..

[17] United Nations Population Fund (2009). State of World Population 2009. Facing a Changing World: Women, Population and Climate.

[18] Institut National de la Statistique et de la Démographie (INSD) (2000). Analyse des Résultats du Recensement Général de la Population et de l’Habitation de 1996.

[19] Fournet F., Meunier-Nikiema A., Salem G. (2008). Ouagadougou: Des Processus D’urbanisation Aux Inégalités Spatiales, Géographie D’une Capitale.

[20] Dos Santos S. (2006). Accès à l’eau et enjeu socio-sanitaire à Ouagadougou. Espace Popul. Soc..

[21] Vallée J., Fournet F., Meyer P.E., Harang M., Pirot F., Salem G. (2007). Stratification de la ville de Ouagadougou (Burkina Faso) à partir d’une image panchromatique Spot 5: Une première étape à la mise en place d’une enquête de santé. Espace Popul. Soc..

[22] Sobngwi E., Mbanya J.C., Unwin N.C., Porcher R., Kengne A.P., Fezeu L., Minkoulou E.M., Tournoux C., Gautier J.F., Aspray T.J. (2004). Exposure over the life course to an urban environment and its relation with obesity, diabetes, and hypertension in rural and urban Cameroon. Int. J. Epidemiol..

[23] Zhao J., Seubsman S., Sleigh A., The Thai Cohort Study Team (2014). Timing of Urbanisation and Cardiovascular Risks in Thailand: Evidence from 51,936 Members of the Thai Cohort Study, 2005–2009. J. Epidemiol..

[24] Teixeira M.G., Costa M.C., Coelho G., Barreto M.L. (2008). Recent shift in age pattern of dengue hemorrhagic fever, Brazil. Emerg. Infect. Dis..

[25] Parker S.P., Cubitt W.D. (1999). The use of the dried blood spot sample in epidemiological studies. J. Clin. Pathol..

[26] Kuno G., Gomez I., Gubler D.J. (1991). An ELISA procedure for the diagnosis of dengue infections. J. Virol. Methods.

[27] Tuntaparsat W., Barbazan P., Nitatpattana N., Rongsryam Y., Yoksan S., Gonzalez J.P. (2003). Seroepidemiological survey among schoolchildren during the 2000–2001 dengue outbreak of Ratchaburi Province, Thailand. Southeast Asian J. Trop. Med. Public Health.

[28] Hosmer D., Lemeshow S. (2000). Applied Logistic Regression.

[29] Meunier-Nikiema A. (2007). Géographie d’une ville à travers la gestion des déchets: Ouagadougou (Burkina Faso). Mappemonde.

[30] Demanou M., Grandadam M., Rogier C., Tolou H., Hervé J.-P., Paupy C., Rousset D. (2009). Séroprévalence de la dengue en milieu urbain au Cameroun. Méd. Trop..

[31] Reiskind M.H., Baisley K.J., Calampa C., Sharp T.W., Watts D.M., Wilson M.L. (2001). Epidemiological and ecological characteristics of past dengue virus infection in Santa Clara, Peru. Trop. Med. Int. Health.

[32] Guha-Sapir D., Schimmer B. (2005). Dengue fever: New paradigms for a changing epidemiology. Emerg. Themes Epidemiol..

[33] Pilon M., Wayack M. (2003). La démocratisation de l’enseignement au Burkina Faso: Que peut-on en dire aujourd’hui?. Cah. d’Études Afr..

[34] Grossman M., Terleckyj N. (1975). The correlation between health and education. Household Production and Consumption.

[35] Bartley L.M., Carabin H., Vinh Chau N., Ho V., Luxemburger C., Hien T.T., Garnett G.P., Farrar J. (2002). Assessment of the factors associated with flavirus seroprevalence in a population in Southern Vietnam. Epidemiol. Infect..

[36] Christophers S.R. (1960). Aedes aegypti (L.) the Yellow Fever Mosquito. Its Life History Bionomics and Structure.

[37] Gubler D.J., Clark G.G. (1995). Dengue/dengue hemorrhagic fever: The emergence of a global health problem. Emerg. Infect. Dis..

[38] Déverin-Kouanda Y. (1993). De la fertilité rurale a la nuisance urbaine. Les difficiles variations culturelles du Tampuure (tas d’ordures) en pays Mossi (Région de Ouagadougou-Burkina Faso). Géogr. Cult..

[39] Arunachalam N., Tana S., Espino F., Kittayapong P., Abeyewickreme W., Wai K.T., Tyagi B.K., Kroeger A., Sommerfeld J., Petzold M. (2010). Eco-bio-social determinants of dengue vector breeding: A multicountry study in urban and periurban Asia. Bull. World Health Organ..

[40] Kamgang B., Happi J.Y., Boisier P., Njiokou F., Hervé J.P., Simard F., Paupy C. (2010). Geographic and ecological distribution of the dengue and chikungunya virus vectors *Aedes aegypti* and *Aedes albopictus* in three major Cameroonian towns. Med. Vet. Entomol..

[41] Cox J., Grillet M.E., Ramos O.M., Amador M., Barrera R. (2007). Habitat segregation of dengue vectors along an urban environmental gradient. Am. J. Trop. Med. Hyg..

[42] Braks M.A., Honorio N.A., Lourenco-De-Oliveira R., Juliano S.A., Lounibos L.P. (2003). Convergent habitat segregation of *Aedes aegypti* and *Aedes albopictus* (Diptera: Culicidae) in southeastern Brazil and Florida. J. Med. Entomol..

[43] Carbajo A.E., Curto S.I., Schweigmann N.J. (2006). Spatial distribution pattern of oviposition in the mosquito *Aedes aegypti* in relation to urbanization in Buenos Aires: Southern fringe bionomics of an introduced vector. Med. Vet. Entomol..

[44] Hayes E.B. (2009). Zika virus outside Africa. Emerg. Infect. Dis..

[45] Institut de Veille Sanitaire (InVS) (2008). Bulletin Hebdomadaire International N°151 6–12 Août 2008.

[46] Russell P.K., Nisalak A., Sukhavachana P., Vivona S. (1967). A plaque reduction test for dengue virus neutralizing antibodies. J. Immunol..

